# Restriction Factor Control of Ty1 Family LTR-Retrotransposons in *Saccharomyces cerevisiae*

**DOI:** 10.3390/v18101154

**Published:** 2026-10-17

**Authors:** Sean L. Beckwith, Mackenzie J. S. Streeter

**Affiliations:** Department of Biology, Hope College, Holland, MI 49423, USA

**Keywords:** Ty1, LTR-retrotransposon, transposable elements, mobile DNA, transposon control, restriction factor, genetic conflict, *Saccharomyces*

## Abstract

Retrotransposons comprise large fractions of many genomes and numerous transposon control mechanisms have evolved to combat retrotransposon expansion. The budding yeast *Saccharomyces cerevisiae* contains several active retrotransposon families but maintains a remarkably compact genome, all without many of the transposon control systems used in mammals. Here, we review restriction factor control of the yeast Ty1 family of LTR-retrotransposons. Several decades of study have identified a novel mechanism that provides highly specific restriction of Ty1 using transposon-derived sequences. Recent work combining structural, biophysical, biochemical, and genetic techniques has revealed a detailed mechanism of this restriction and the determinants of specificity. Interestingly, two Ty1 subfamilies each converged on the same restriction mechanism despite following separate evolutionary histories. The protein p22/p18 is a self-encoded restriction factor that targets Ty1c and is produced from an internally initiated mRNA. Drt2 is a restriction factor that targets Ty1′ and is produced from a transposon fragment endogenized by the host genome. Both interfere with the assembly of transposon virus-like particles using a shared dimerization surface, but subtle sequence divergence in this interface directs each restriction factor against its specific Ty1 subfamily. This work presents a framework to identify novel transposon control systems in other organisms.

## 1. Introduction

The budding yeast *Saccharomyces cerevisiae* was the first eukaryotic genome sequenced 30 years ago [1,2] and now the genomes of more than 1000 *S. cerevisiae* strains spanning the species’ phylogenetic tree have been collected [3] and provide a near telomere-to-telomere pangenome view of genome evolution [4,5]. Decades of sustained interest have kept yeast on the cutting edge as a model for understanding the dynamics of eukaryotic genomes on scales ranging from milliseconds to millennia. It is no surprise, then, that yeast has also provided detailed insights into the replication cycles of some of the most pervasive and prominent forces of genome evolution: transposable elements. These mobile genetic elements, first described by Barbara McClintock more than seven decades ago [6,7,8], are found in all branches of life, exhibit considerable sequence diversity, and vary wildly in abundance across diverse genomes [9]. Transposons in eukaryotes are classified based on their mode of replication: retrotransposons use an RNA-intermediate that is reverse transcribed and integrated (referred to as a “copy-and-paste” mechanism) whereas DNA transposons typically excise the element itself and mobilize it to a new genomic location (referred to as a “cut-and-paste” mechanism) [10]. Retrotransposons are further divided into subclasses based on the mechanism of replication and integration. For instance, long terminal repeat (LTR)-retrotransposons perform the reverse transcription reaction in the cytoplasm encased within a capsid assembled by their Gag protein [9,11,12].

Transposon replication is a major driver of genome size and accounts for large fractions of many plant genomes [13,14] and the giant genomes of salamanders [15,16,17]. Transposons are an incredible reservoir of raw sequence material for evolution. The majority of *cis*-regulatory DNA in the human genome is derived from transposon sequences [18,19]. There are also numerous examples of transposon proteins that have been co-opted in mammals for host physiology and development [20,21,22,23,24]. However, new transposon insertions can have deleterious effects on genome integrity and are implicated in various human diseases including cancer, Alzheimer’s, inflammaging, and others [25,26,27,28,29,30]. Therefore, a number of transposon control mechanisms have evolved, many of which also act on exogenous viruses [31].

Several enzymes that modify DNA, regulate DNA synthesis, or modulate nucleic acid degradation act as restriction factors against transposons and retroviruses, which are related to retrotransposons. RNA interference (RNAi) is an ancient strategy found in plants, animals, and fungi that can restrict transposon and viral replication. Transposon or viral sources of RNA are processed into short interfering RNAs (siRNAs) by Dicer and loaded into the RNA-induced silencing complex (RISC) which then uses its catalytic core consisting of Argonaute proteins to cleave complementary sequences in the transposon or virus [32]. Piwi-interacting RNAs (piRNAs) work with Piwi family Argonaute proteins to limit transposon activity in animal germlines independent from Dicer [33]. Moloney leukemia virus 10 (MOV10) protein was first identified in mice [34] and is a homolog of RNA helicases involved in RNAi pathways in *Arabidopsis* and *Drosophila* [35,36]. In humans, MOV10 inhibits retroviruses and retrotransposons, interacting with the human non-LTR-retrotransposon Long Interspersed Nuclear Element-1 (LINE-1) by liquid–liquid phase separation leading to RNA degradation [37,38,39]. The activation-induced cytidine deaminase/apolipoprotein B mRNA editing enzyme catalytic polypeptide-like (AID/APOBEC) family of cytosine deaminases restrict human immunodeficiency virus-1 (HIV-1) replication by deaminating cytosines into uracil [40]. This family is generally conserved in vertebrates and the *APOBEC3* genes have greatly expanded in placental mammals [41]. To restrict HIV-1 replication these enzymes can become encapsidated into virions where minus strand DNA synthesized by reverse transcriptase from the genomic HIV RNA is vulnerable to APOBEC deamination of cytosines to uracil [42,43,44]. This induces C/G to T/A mutations which functionally inactivate the virus or result in its degradation through subsequent DNA repair pathways [40,45]. Mammalian sterile alpha motif and histidine-aspartic acid (HD)-domain-containing protein 1 (SAMHD1) is a deoxynucleoside triphosphate triphosphohydrolase that depletes the intracellular pool of dNTPs to levels that cannot support reverse transcription of HIV-1 and is a potent inhibitor of LINE-1 [46,47,48,49,50,51].

*S. cerevisiae* contains several families of retrotransposons yet does not utilize RNAi and none of the restriction factors described above have been identified as restrictors of retrotransposition in *S. cerevisiae*. Given the potential for retrotransposons to greatly expand genome size, it is remarkable that yeast maintains such a compact genome. Ty1 is an LTR-retrotransposon in *S. cerevisiae*, discovered more than 40 years ago, that replicates through an RNA-intermediate reverse transcribed in cytoplasmic virus-like particles (VLPs) [52,53]. Ty1 has been a powerful model to understand mechanistic details of retrotransposon and retroviral replication cycles. This review describes a transposon control mechanism that targets elements of the yeast Ty1 family. We briefly introduce Ty1 and then detail the discovery, characterization, and evolution of transposon control in two related Ty1 subfamilies.

## 2. The Ty1 LTR-Retrotransposons of *S. cerevisiae*

*S. cerevisiae* harbors several families of LTR-retrotransposons including members of *Pseudoviridae* (Ty1, Ty2, Ty4, Ty5) and *Metaviridae* (Ty3) [54,55,56]. Ty1 is a family with active elements and has been a longstanding model for understanding retrotransposon biology. For more thorough reviews on Ty1 beyond transposon control, we refer the reader to prior publications [12,57,58,59,60]. Despite lacking the mammalian transposon control mechanisms mentioned above, transposons comprise only a small fraction of the *Saccharomyces* genome. Instead, excessive retrotransposition of Ty1 family elements is prevented by the restriction factors p22/p18, Drt2, and perhaps others. Here, we describe Ty1 elements in laboratory and wild strains, highlight key steps in the Ty1 replication cycle, and briefly review host interactions.

### 2.1. Ty1 Elements in Laboratory and Wild Strains

**Figure 1:**
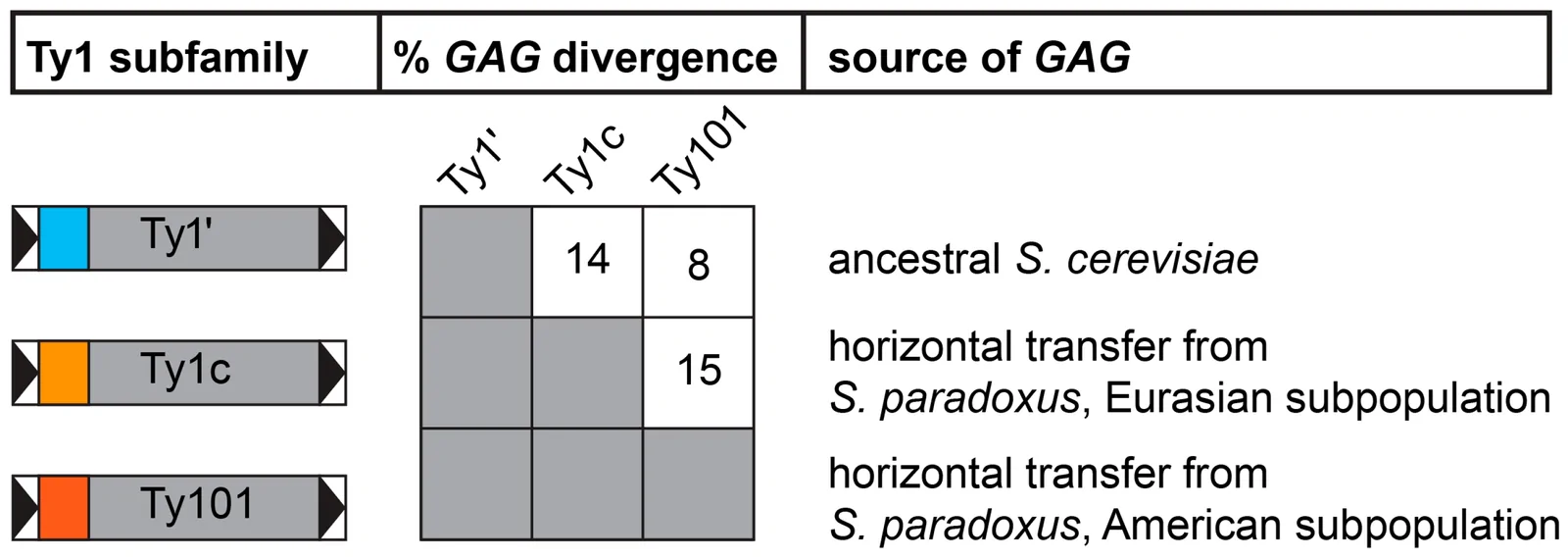
Ty1 subfamily classifications in *S. cerevisiae*. Three subfamilies of Ty1 have been described in *S. cerevisiae*: Ty1′, Ty1c, and Ty101. The nucleotide sequence divergence of *GAG* between each subfamily and the source of the *GAG* gene is shown; the colored regions in the Ty1 subfamily schematics represent the *GAG* sources. Data from reference [55]; see reference [55] for further information on sequence variants, mosaic structure, and prevalence of each subfamily.

A limited number of yeast strains have been used to study Ty1 mechanistically but recent pangenome sequencing efforts have cataloged the content of Ty1-Ty5 across a wide array of strains [4,5]. The copy number of Ty elements varies considerably, ranging from 0–120 complete elements per genome with a median number of 14.5 [4]. The Ty1 family includes three subfamilies in *S. cerevisiae*: canonical Ty1 (Ty1c), Ty1′, and Ty101 (Figure 1) [55]. These Ty1 subfamilies are differentiated based on sequence divergence, prominently in *GAG* due to the distinct origins of their *GAG* genes: Ty1′ is the ancestral element in *S. cerevisiae* and Ty1c and Ty101 have arisen from horizontal transfer events from *S. paradoxus*, the sister-species of *S. cerevisiae* [55,61]. Ty1′ is the most widely distributed subfamily and is typically present at low copy number whereas Ty1c is the most abundant subfamily but is limited to certain isolates [55]. The distribution of Ty101 is much more restricted and has been detected primarily in lineages from Central and South America [55]. The reference strain S288C contains 35 full-length copies of Ty1c and 3 full-length copies of Ty1′; copy number estimates have varied based on sequencing technologies but long-read PacBio assemblies are the most accurate to date [54,61,62]. However, to avoid contributions by endogenous Ty1 elements, non-reference *Saccharomyces* strains lacking full-length Ty1 elements have been particularly useful in the study of restriction mechanisms. The first strain (337 and its derivative DG1768), which was originally thought to be *S. cerevisiae* [63] but subsequently determined to be a strain of *S. paradoxus* [64,65], has been used in the majority of studies characterizing Ty1c copy number control (CNC) [66,67,68,69,70,71]. A second strain is a Ty1-less *S. cerevisiae* (UWOPS05-227.2) that has been used in the identification of the Ty1′ restriction factor [72]. Much of the Ty1c work has characterized restriction of a recombinant element called Ty1H3 (GenBank M18706.1) (originally called TyH3) that was captured by insertion into a plasmid from a derivative of S288C called JB84A [52,54,73]. Ty1′ restriction has been characterized for a model element (YBLWTy1-1) taken from S228C [72]. Throughout this review, we will refer to Ty1 when describing general knowledge about the family (most of which has been elucidated using Ty1c and in particular Ty1H3) and Ty1c or Ty1′ for subfamily-specific findings.

### 2.2. The Ty1 Genome and Replication Cycle

**Figure 2:**
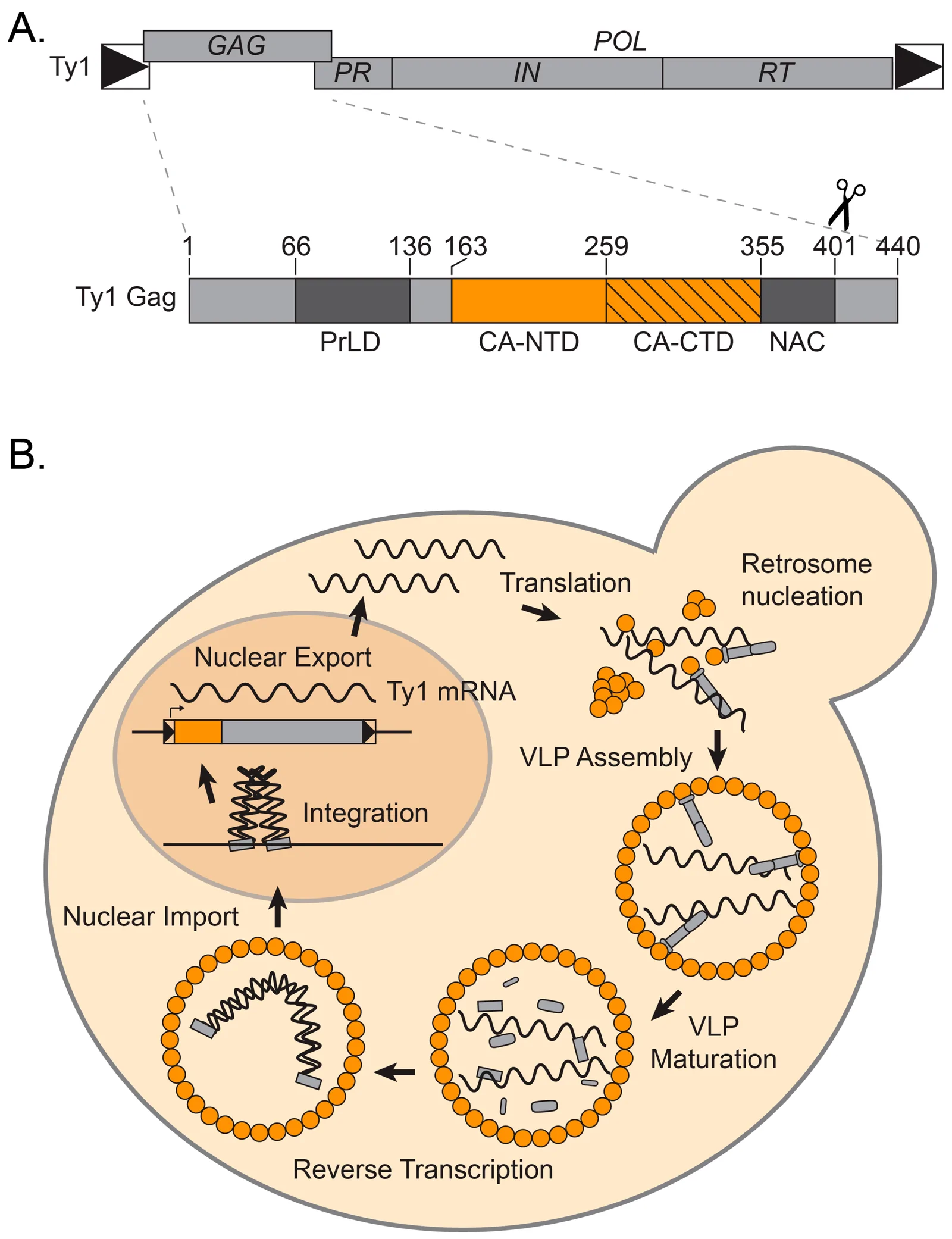
Genome organization and replication cycle of Ty1. (**A**) The Ty1 genome contains 5′ and 3′ long terminal repeats (boxed arrowheads) flanking two overlapping open reading frames. *POL* encodes a polyprotein that is post-translationally cleaved into separate protease (PR), integrase (IN), and reverse transcriptase-RNase H (RT) proteins. For the *GAG* gene, a detailed view of protein domains is presented (PrLD: prion-like domain, CA-NTD: capsid N-terminal domain, CA-CTD: capsid C-terminal domain, NAC: nucleic acid chaperone), the CA-CTD is hatched to represent the restriction factor sequence. The proteolytic cleavage site at amino acid 401 is represented by scissors. (**B**) Major steps of the Ty1 replication cycle are illustrated. Transcription of a Ty1 element in a host chromosome is followed by nuclear export and translation. Cytoplasmic retrosomes are nucleated as foci of Ty1 proteins and RNA which serve as the assembly sites of VLPs. Proteolytic maturation occurs within VLPs permitting reverse transcription of the RNA genome. The Ty1 preintegration complex is imported into the nucleus where integrase binds RNA Polymerase III and the cDNA is integrated into a host chromosome resulting in an increase in Ty1 copy number.

Ty1 elements have an analogous genome organization to retroviruses but lack an envelope gene; two overlapping open reading frames encode Gag and Pol proteins [12] (Figure 2). Pol is a polyprotein that is post-translationally cleaved into separate protease (PR), integrase (IN), and reverse transcriptase-RNase H (RT) proteins. Gag contains a prion-like domain (PrLD), a capsid domain (CA) that is structured similarly to retroviral capsid with a capsid N-terminal domain (CA-NTD) and a capsid C-terminal domain (CA-CTD), and a nucleic acid chaperone domain (NAC) (Figure 2). Following gene expression, both the Ty1 mRNA and proteins colocalize in cytoplasmic foci called retrosomes [74,75,76]. Retrosomes require the PrLD to self-aggregate and are hypothesized to be liquid–liquid phase-separated membraneless organelles that serve as the assembly site of VLPs [77,78]. VLPs are assembled as a lattice of CA and encapsidate the Ty1 proteins and Ty1 mRNA bound by NAC. Ty1 protein maturation by PR and reverse transcription by RT occur within VLPs and the Ty1 cDNA and IN are imported into the nucleus [12]. Ty1 has a strong insertion site preference predominately upstream of tRNA genes due to a physical interaction with RNA Polymerase III [79,80,81,82].

### 2.3. Interactions Between Ty1 and the Host

*S. cerevisiae* and Ty1 have co-evolved in ways that are frequently mutually beneficial. RNA Polymerase III-tethered insertion of Ty1 elements results in “safe havens” where insertion rarely disrupts genes and causes minimal harm. Ty1 insertions can alter expression of neighboring genes and generate segmental duplications, each of which can promote adaptation to various stresses [83,84,85,86,87,88,89]. Furthermore, a variety of host genes modulate several steps of the Ty1 replication cycle [12,90,91,92,93,94,95,96,97,98]. On the whole, Ty1 is beneficial enough to survive in natural populations and laboratory evolution experiments [55,63,86,88,89,99]. Excessive retrotransposition can be deleterious [100], so the balance maintained by the collective output of these host genes and the transposon-derived proteins described in the following sections is essential for *S. cerevisiae* to coexist with its “tamed” Ty1 retrotransposon.

## 3. Copy Number Control of Canonical Ty1

The copy number of Ty1 elements and other LTR-retrotransposons in laboratory and natural yeasts is the result of element gain either by new insertions or recombination of cDNA (collectively “retromobility”) balanced against element loss either by LTR-LTR recombination or mutation and sequence decay. Here, we briefly review the initial studies of transposon control of Ty1 before describing the discovery and mechanistic dissection of the p22/p18 restriction factor protein that targets Ty1c.

### 3.1. Initial Studies of Restriction of Ty1 Retromobility

Two mechanisms of Ty1c CNC were described in the early 2000s. Transcriptional cosuppression was described as a mechanism that inhibited Ty1c RNA accumulation due to the overproduction of a non-translatable mRNA [101,102,103]. However, the overproduction of non-translatable mRNA had no impact on Ty1c retromobility [101].

A post-transcriptional mechanism of CNC was described shortly after the initial report of transcriptional cosuppression [66]. Here, increasing the chromosomal copy number of Ty1c elements increased RNA accumulation, but paradoxically, decreased retromobility levels. Further, a possible role for antisense RNAs from the 5′ region of Ty1c was identified that both transcriptionally silenced Ty1 and decreased retromobility [104,105]. However, ectopically expressed antisense RNAs failed to inhibit in trans, undermining a direct role for antisense RNAs in restricting retromobility [105].

### 3.2. Self-Encoded Copy Number Control of Canonical Ty1

The report of a cryptic promoter in 2015 that initiated an internal sense RNA translated in the same reading frame as Gag [67] was the watershed discovery that has led to a molecular understanding of Ty1c restriction. This finding and a series of subsequent studies have already been reviewed [106,107,108]; here, we first summarize those data and then review recent work combining structural, biophysical, biochemical, and genetic approaches to detail the mechanism of restriction of Ty1c.

#### 3.2.1. Identifying the Restriction Factor

The Ty1c mRNA does not encode the restriction factor as evidenced by CNC in strains containing a silenced Ty1c on a multicopy plasmid [66,105]. Here, plasmid-borne Ty1c expression was under the control of the yeast *GAL1* promoter, which is induced in the presence of galactose but repressed in the presence of glucose. Strains grown in glucose-containing media had Ty1c expression from the plasmid repressed but a decrease was still observed in the levels of retromobility of a chromosomal Ty1c element under the expression control of its native LTR promoter and marked with a retromobility indicator gene. These results suggested a role for an inhibitory noncoding RNA or restriction protein produced from a subgenomic transcript, prompting the investigations of Ty1c antisense RNAs described above. However, studies using Selective 2′ Hydroxyl Acylation analyzed by Primer Extension (SHAPE) and other techniques revealed that antisense RNAs do not anneal with Ty1 genomic RNA and are not packaged in the VLP [109]. Although antisense RNAs did not fully explain CNC hallmarks including low levels of mature IN and RT protein products, Ty1c cDNA accumulation, and reduced retromobility, a trans-acting restriction factor that works post-translationally would.

A genetic screen for mutants lacking CNC, northern blotting, and cap-independent 5′ Rapid Amplification of cDNA Ends (RACE) analysis mapped a subgenomic sense-strand RNA termed Ty1i with a 5′ terminus at nucleotide 1000, 760 bases downstream of the Ty1c mRNA [67]. This transcript initiates from within the *GAG* gene and is translated in the same reading frame as Gag. The protein produced from Ty1i, p22, is identical to the C-terminal half of Gag-p49 and is processed by PR to produce p18, which is identical to the C-terminal half of mature Gag-p45. Generating custom antisera, and later a commercially available polyclonal antibody (Boster Bio cat. no. DZ33975), allowed the detection of p22/p18 protein produced from multiple chromosomal elements [67]. Subsequent work using epitope-tagged p22/p18 has facilitated western blot analyses of mutated p22/p18 sequences [68,70]. Because Ty1i is self-encoded, Ty1c CNC is due to a progressive increase in the levels of p22/p18 produced by increasing copy numbers of Ty1c elements in the genome.

#### 3.2.2. Restriction Factor Expression

Ty1i contains two alternative start codons, AUG1 and AUG2, located at the 5′ end of the Ty1i transcript. Mutational analyses of plasmid-borne Ty1c constructs reveal that either AUG1 or AUG2 can be used to synthesize p22/18 proteins [69]; however, Ribo-seq reads support initiation at AUG1 [67,110] and AUG1 protein products are produced at a much higher level than AUG2 when expressed under the same *GAL1* promoter on a plasmid [68]. These additional ten amino acids translated from AUG1 do not appear in X-ray crystallography electron density maps and, while they appear not to be structured, may stabilize the protein [68]. Collectively, these data imply that a restriction factor initiated at either AUG1 or AUG2 can inhibit Ty1c retromobility, but that AUG1 is the functional product generated in cells.

Ty1c mRNA and Ty1i RNA are regulated by different classes of yeast promoter architecture [111,112]. Approximately 90% of yeast promoters do not contain TATA sequences, are TFIID-dependent, and are usually more constitutively active. By contrast, around 10% of yeast promoters are dependent on SAGA and contain TATA boxes; these promoters are highly regulated and are enriched in stress-response genes [113]. The full-length Ty1c mRNA is initiated in the 5′ LTR, the promoter sequence contains a TATA box, and transcription is SAGA-dependent. The Ty1i promoter, instead, is TATA-less and TFIID-dependent.

The ratio between Ty1c mRNA and Ty1i RNA is controlled by the host through the positioning of Mediator binding [106,111]. Mediator is an evolutionarily conserved co-activator complex found in all characterized eukaryotes that promotes transcription of RNA Polymerase II genes. The core Mediator complex is composed of three distinct modules—head, middle, and tail. Mutants of the different modules have distinct cis-acting effects on Ty1i RNA levels and retromobility. The tail module promotes Mediator occupancy at the native promoter of full-length Ty1c in the 5′ LTR rather than the internal Ty1i promoter. Deletion of tail module subunits increases Ty1i RNA and p22 protein levels, and reduces retromobility. Deletion of a head subunit eliminated Mediator occupancy at the Ty1i promoter [111].

### 3.3. The Mechanism of Restriction of Ty1c

Insights into the inhibitory mechanism of p22/p18 have come through a combination of genetic, biochemical, biophysical, and structural studies. A forward genetic screen identified mutations in Gag that allow retromobility in the presence of p22/p18, suggesting that the target of p22/p18 is Gag [70]. Resistance mutations cluster in two regions of Gag: (1) the CNC^R^ region spanning Gag residues 170–220, which falls within the CA-NTD [114], and (2) what was previously called the UBN2/Retrotran_gag_2 domain based on sequence homology and is now described based on structural data as the CA-CTD [68]. In pulldown experiments in yeast, p18 interacted with Gag expressed from multiple endogenous Ty1c elements [67] and with the C-terminal half of Gag expressed from a plasmid [69]. p18 also fractionated with Ty1 VLPs and changed the sedimentation pattern of Gag, indicating altered VLP assembly products [67,70]. *LOC1*, a gene involved in ribosome biogenesis and regulation of yeast mating-type switching [115,116], was identified as a CNC-specific Ty1c cofactor [117]. Deletion of *LOC1* increased p22 protein levels and this disrupted Gag multimerization, raising the possibility that p22 interferes with early stages of VLP assembly [117].

#### 3.3.1. Structural Insights into the Restriction Mechanism

**Figure 3:**
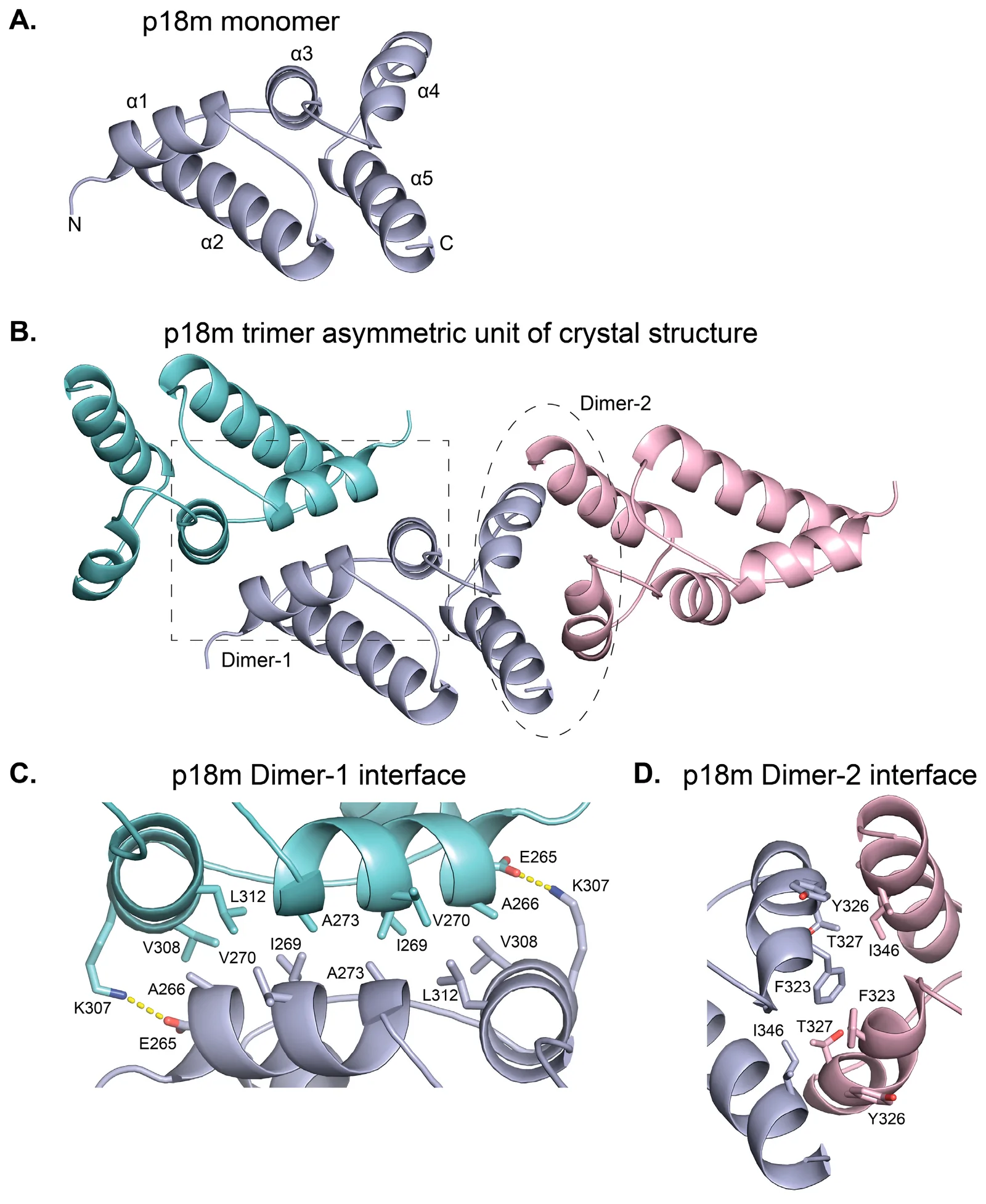
Crystal structure of the p18m restriction factor. (**A**) Crystal structure of p18m monomer; the protein backbone is shown in cartoon representation. The five α-helices in the structure are labeled sequentially from the N- to C-terminus. (**B**) The trimer asymmetric unit of the p18m crystal structure. The three p18m protomers are shown in cartoon representation colored cyan, light blue and pink, respectively. The dimer interfaces between protomers (Dimer-1 and Dimer-2) are highlighted with the gray box and ellipse, respectively. (**C**) Close-up view of the Dimer-1 interface, the box-highlighted region in (**B**). Residues on helices α1 and α3 that make apolar and salt bridge interactions are shown in stick representation. The E265 and K307 salt bridge is indicated by the dashed lines. (**D**) Close-up view of the Dimer-2 interface, the ellipse-highlighted region in (**B**). Residues on helices α4 and α5 that make the hydrophobic interactions are shown in stick representation. Figure adapted from Figure 2 of reference [68] with added labels identifying each structure and omitting the panel presenting the alignment of monomers.

At the time of the discovery of Ty1i and p22 in 2015, structural studies of Ty1c Gag and VLPs had not been published since the 1990s [118,119,120]. Significant technical advancements have been made and recent efforts have contributed high-resolution crystallographic [68,71] and cryo-electron tomography data [114]. Work published in 2021 defined a minimal fragment of the p22/p18 restriction factor called p18m, demonstrating that the nucleic acid chaperone domain of Gag was not required for restriction [68]. p18m constructs expressed from both AUG1 and AUG2 were purified from *E. coli* and the structure was solved by X-ray crystallography at 2.8 Å and 3.12 Å resolution, respectively [68]. In the descriptions that follow, amino acid coordinates refer to positions within the full-length Gag protein. p18m is highly structured, forming a five-helix bundle with electron density spanning residues D262-Q351 (Figure 3). The structure reveals that p18m is a CA-CTD that is highly related to the CA-CTDs of several retroelements. Top similarity hits included the *Drosophila* and mammalian ARC proteins (an exapted *GAG* gene), the yeast Ty3 CA, and the CA-CTD of the gamma-retroviruses murine leukemia virus (MLV). Both CA-CTDs and CA-NTDs provided strong matches, supporting the hypothesis that tandem CA-NTD and CA-CTD arose as a gene duplication event [121]. Weaker but significant hits included HIV-1 CA and the human endogenous retrovirus HERV-K [68]. These similarities demonstrate the evolutionary relationships between retrotransposons and retroviruses.

Structural and biophysical analyses of p18m also suggested a restriction mechanism. The asymmetric subunit of the crystal structures consists of three monomers that each form two dimer interfaces (Dimer-1 and Dimer-2) [68]. Dimer-1 comprises an extensive hydrophobic interface burying 773 Å^2^ of helixes 1 and 3 of two opposing monomers with important contributions from the sidechains of A266, I269, V270 A273, I302, I304, V308, and L312. Dimer-1 is further stabilized by salt bridges between E265 and K307 on the edges of this continuous apolar network. The structures of ARC and retroviral CA-CTDs also contain an equivalent hydrophobic core. Dimer-2 is a second hydrophobic interface that packs helixes 4 and 5 (including residues F323, Y326, T327, A345, and I346) of opposing monomers but is not sequence conserved in Ty3, ARC, or retroviral CA. While p18m packed as a trimer to form the basic repeating unit of the crystal under conditions promoting crystallization, to determine p18m oligomerization in solution, size exclusion chromatography and analytical ultracentrifugation experiments were performed. These showed that p18m is a stable obligate dimer and can form higher-order structures in solution. Sedimentation equilibrium data determined *K_D_* values of 0.73 µM for the tightly associating monomer-dimer equilibrium and 43.2 µM for the weakly associating dimer-tetramer equilibrium [68].

#### 3.3.2. The Binding Interface Targeted by p22/p18 Restriction

**Figure 4:**
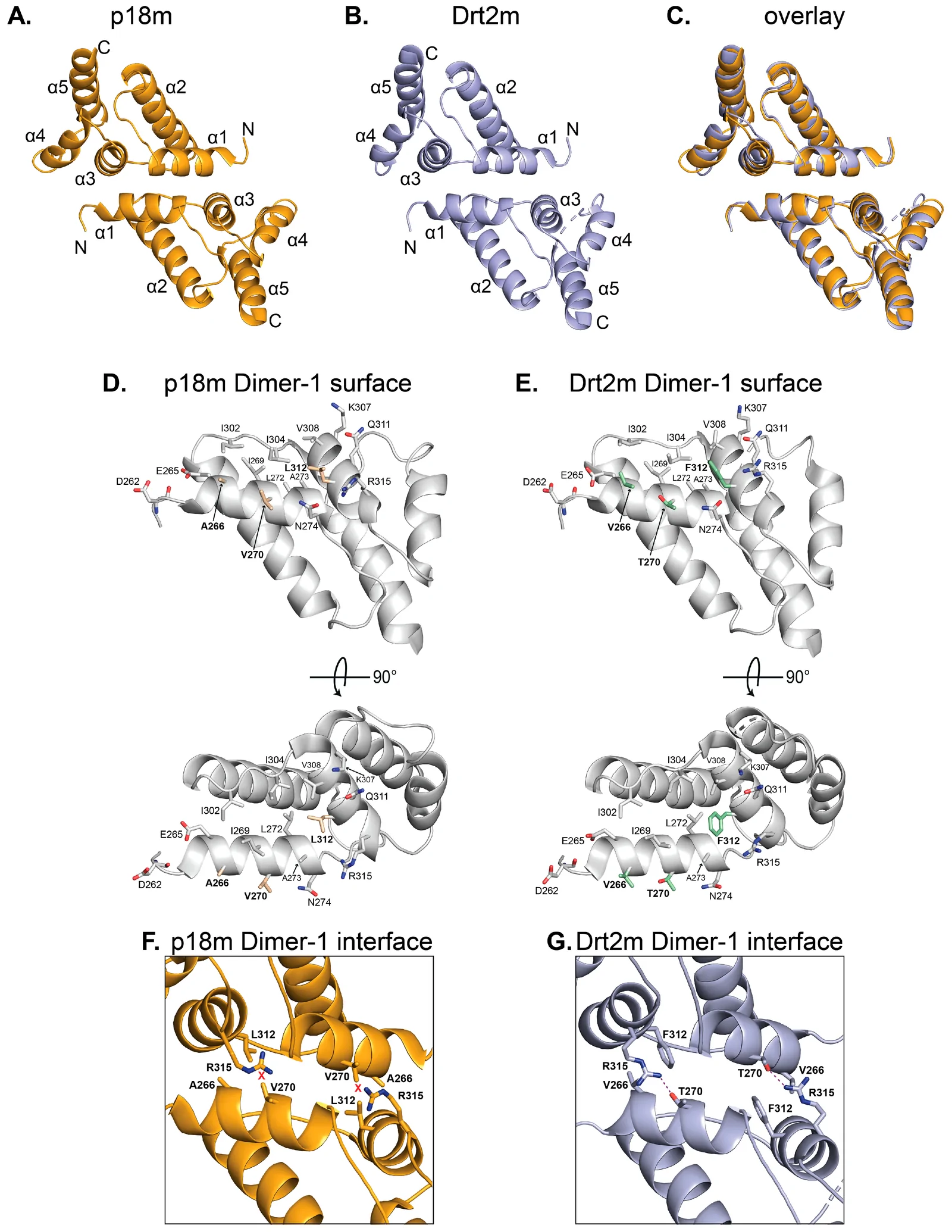
Crystal structures of p18m and Drt2m restriction factors. Crystal structures of (**A**) p18m and (**B**) Drt2m dimers. The protein backbone is shown in cartoon representation, p18m in orange and Drt2m in pale blue. Helical secondary structures in each monomer are labeled sequentially from N- to C-terminus. (**C**) 3D structural superposition of p18m and Drt2m dimers. Coloring and orientation are the same as in A and B. (**D**) p18m and (**E**) Drt2m Dimer-1 surfaces. The view is perpendicular to (upper) and into (lower) the interface of a single monomer. The protein mainchain is shown in gray cartoon. Residues that make apolar and salt bridge interactions at the interface are shown in stick representation. Specificity determining residues in p18m (A266, V270 and L312) and Drt2m (V266, T270 and F312) are highlighted with bold labeling and shown in light brown and green respectively. View of the Dimer-1 interface of (**F**) p18m and (**G**) Drt2m. View and cartoon coloring are the same as in A and B. The variable interfacial residues (A/V)266, (V/T)270 and (L/F)312 together with R315 on helices α1 and α3 are shown in stick representation. The T270-R315 hydrogen bonding interaction at the Drt2m interface is indicated by the purple dashes. Red crosses indicate a lack of hydrogen bonding in p18m interface. Figure adapted from Figure 2 of reference [71] with added labels identifying each structure.

These in vitro data suggested that p18m restriction may operate by forming heterodimers between identical sequences in p18m and Gag using Dimer-1 and/or Dimer-2 (Figure 4). Genetic and biochemical experiments in yeast were used to test this hypothesis. Targeted mutagenesis of Dimer-2 residue F323 did not affect protein expression but suppressed higher-order oligomerization in solution [68]. Restriction activity was not impaired in these mutants, implying that the mechanism of restriction utilizes dimerization mediated by Dimer-1. However, the same mutations in Gag markedly reduced retromobility without reducing protein expression. These Dimer-2 data identify separation-of-function mutants in which F323 is important for Gag function in Ty1c retromobility but dispensable for p18m function in CNC [68,71]. By contrast, mutational analyses demonstrated that Dimer-1 is critical for p18m integrity and function. Mutations were created in Dimer-1 both by targeted mutagenesis and using an NNK codon library (N = A/C/T/G, K = G/T) which randomizes all 20 amino acid codons while reducing premature stop codons. Conservative mutations were tolerated but mutations that disrupt the Dimer-1 interface resulted in loss of protein levels and restriction [68]. Ultracentrifugation through sucrose gradients can be used to fractionate yeast lysates to test the effects of p22 and p18m mutants on VLP assembly products [67,68,70,71]. p22 and p18m mutants that restrict Ty1c co-fractionate with VLPs and induce a striking redistribution of Gag toward the top of the sedimentation gradient, consistent with incompletely assembled VLPs.

Formation of heterodimers between p18m and Gag was then directly tested in vitro using recombinant proteins. Much of the in vitro work was performed on constructs containing the F323S mutation that disrupts the Dimer-2 interface and suppresses higher order aggregation in order to facilitate purification and chromatographic analysis [68,71]. Purified p18m-F323S was mixed with a purified CA domain of Gag-F323S (residues 169–355). Each purifies separately as homodimers; the two are then mixed, incubated for 20 h, and the level of exchange to form heterodimers is measured using size exclusion chromatography coupled with multi-angle laser light scattering (SEC-MALLS). p18m and CA formed a large proportion of heterodimers indicating a high level of exchange and compatibility between the identical Dimer-1 interface present in both proteins [71].

#### 3.3.3. A Lattice-Poisoning Model of Restriction

To further probe the model that p22/p18 restricts Ty1c by interfering with Gag-Gag interactions required to assemble complete VLPs, the expression kinetics of p18m and Ty1c were unlinked. The relative level and timing of expression of Ty1c and the restriction factor matters for restriction potency. Particular mutants located within Dimer-1 have shed light on these kinetic parameters. The Gag-A273V mutant allows plasmid-borne Ty1c overexpressed under the galactose-inducible *GAL1* promoter to escape restriction by p22 produced from multiple wildtype chromosomal Ty1c copies [70]. However, when simultaneously overexpressed, p18m-A273V restricts wildtype Ty1c as well as wildtype p18m does [68]. To uncouple Ty1c and p18m expression, a single endogenously expressed chromosomal Ty1c element was challenged with inducible plasmid-borne p18m. Ty1c-A273V partially escaped restriction from wildtype p18m and, similarly, wildtype Ty1c partially escaped restriction from p18m-A273V [68].

**Figure 5:**
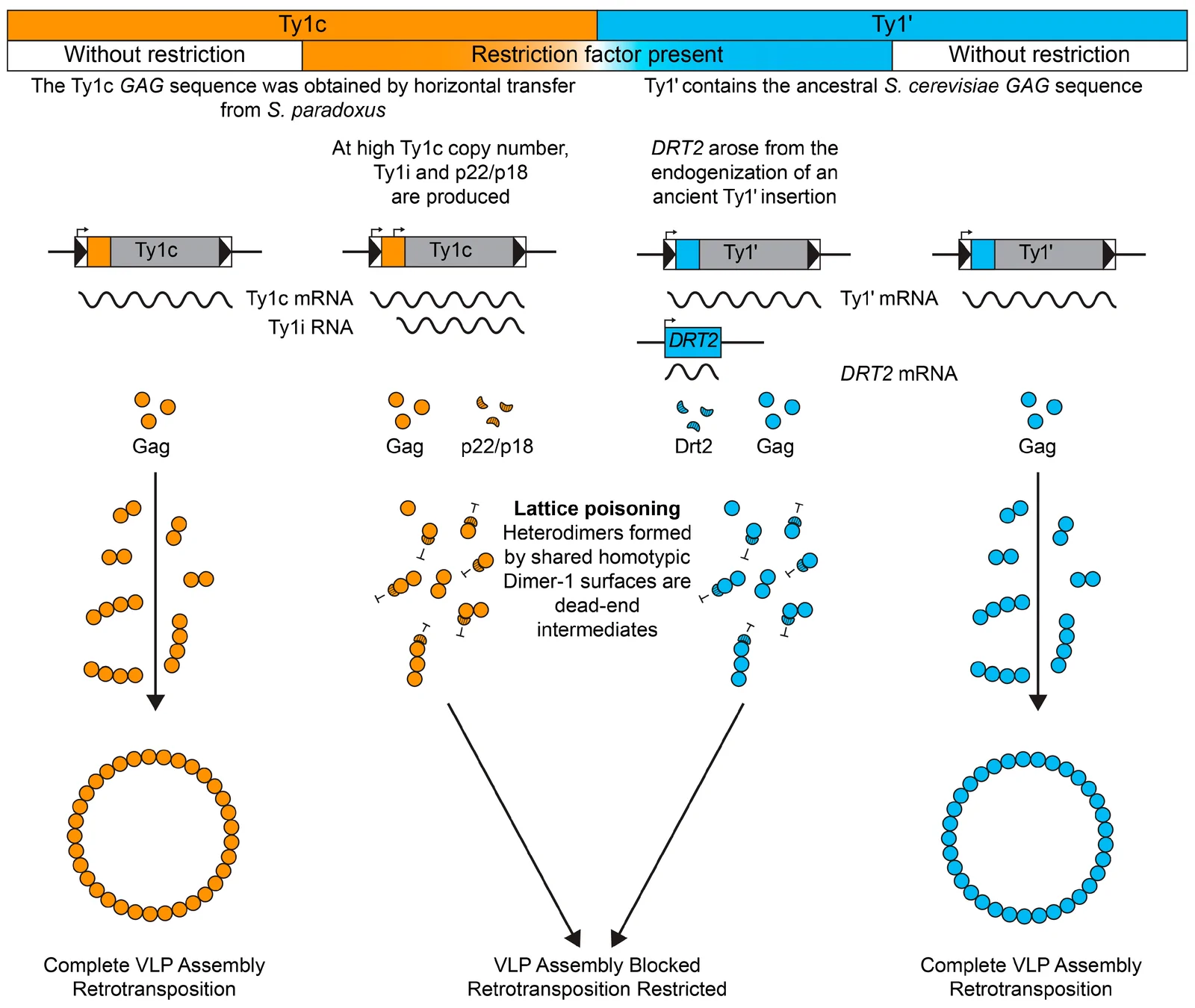
The lattice-poisoning model of restriction of Ty1c and Ty1′ elements in *S. cerevisiae*. In the absence of restriction factor expression, Gag proteins assemble into complete VLPs which permit retrotransposition. The presence of p22/p18 or Drt2 restriction factors blocks VLP assembly and therefore retrotransposition. p22/p18 is produced from an internally initiated Ty1i RNA and Drt2 is expressed from an endogenized *GAG* gene at a fixed chromosomal locus. Each restriction factor binds to its respective Gag protein using homotypic surfaces at the shared Dimer-1 interface forming heterodimers that are dead-end intermediates, thus poisoning the assembling VLP lattice.

These data have led to a model for restriction of Ty1c by p22/p18 (Figure 5). Given that p18m is identical to the CA-CTD of Gag and that capsid assembly involves interactions between hundreds of copies of Gag, binding of p18m with the CA-CTD of Gag will interfere with lattice assembly of the Ty1c VLP. Because p18m is a CA-CTD without the CA-NTD, these p18m-Gag heterodimers are dead-end intermediates that block complete VLP assembly. The range of assembly intermediates leads to the observed broad distribution of Gag sedimentated through sucrose gradients [67,68,70,71]. Mutations that disrupt homotypic pairing between p18m-Gag at the Dimer-1 interface prevent efficient restriction from occurring [68,70,71]. This model predicts that p22/p18 can inhibit Ty1c retromobility at low stoichiometries because not every Gag-Gag interaction need be inhibited to poison assembly of a complete VLP lattice. However, it also predicts that inhibition by p22/p18 must occur relatively early in the VLP assembly pathway and that pre-formed Gag complexes will be resistant to p22/p18. Chromosomal Ty1c expressed constitutively from its native promoter is inhibited at a much lower level by galactose-induced p18m than Ty1c on a plasmid that is simultaneously overexpressed with p18m [68]. The continued study of the dynamics between p22/p18 and Ty1c that occur naturally will be an interesting avenue of exploration.

## 4. Restriction of the Ty1′ Subfamily

Ty1c has been intensely studied and has contributed immensely to our mechanistic understanding of retrotransposon biology [12]. It is, however, only one of the LTR-retrotransposons found in the yeast Ty1 family. Ty1′ is a closely related subfamily to Ty1c that is also found in the S288C reference strain of *S. cerevisiae* and is widely distributed [54,55,61]. Unlike Ty1c, Ty1′ retromobility increases with copy number [72]. This demonstrated that Ty1′ does not have a self-encoded restriction factor like p22/p18 that confers CNC and no evidence has been found of a Ty1i-like RNA produced from Ty1′. Instead, Ty1′ is under transposon control by a recently described host gene called *DRT2* (domesticated restriction of Ty1′ relic 2) [72].

### 4.1. Phylogenomics of the DRT2 Locus

*DRT2* is found at a fixed locus on chromosome IV in a wide distribution of *S. cerevisiae* lineages but is not present in S288C, highlighting the value of studying non-reference strains [56,61,72]. Detailed analysis of the evolutionary history and diversity of *DRT2* was performed using de novo local assembly of the locus from 73 nonredundant high-quality long-read whole genome sequencing data sets [72]. The resulting maximum likelihood phylogeny was rooted using a clade containing strains from the deepest ancestral lineage of *S. cerevisiae* (the China IX/Taiwanese lineage) [3,122]. *DRT2* is widely distributed and is found in French dairy, Asian fermentation, West African, North American oak, most Chinese, and several mosaic lineages. Its presence in the most ancient lineages and in all wild lineages except European oak and China I indicates that *DRT2* was likely present in the common ancestor of all global *S. cerevisiae* lineages. There have been three loss events inferred including in the lineage leading to the clade of European wine, European oak, and the reference strain S288C [72]. S288C has a complex genealogy that includes numerous deliberate crosses [2]. A fourth loss is an apparent gene conversion event with a Ty101 element in an ancestor of a South American clade.

The structure and content of the *DRT2* locus have considerable variation but the coding region of *DRT2* has been quite well conserved, indicating its functional importance for the yeast in restricting Ty1′ retromobility. The locus is between two tRNA genes oriented on opposite strands with *DRT2* residing upstream of both. Based on our understanding of Ty1c insertion site targeting to regions upstream of tRNA genes due to a physical interaction with two subunits of RNA Polymerase III [79,80,81], the *DRT2* locus is a hotspot for transposon activity. The *S. cerevisiae* strains examined, regardless of whether they contain *DRT2*, have a variety of truncated and full-length Ty1 elements, Ty2 elements, and solo LTR remnants from Ty1, Ty2, Ty3, and Ty4 at this locus [72]. Elements and their fragments are observed in both orientations and in several instances a full-length element has inserted into a decayed element. These showcase that there have been successive gain and loss events of numerous Ty elements over time, making the locus an ideal place for the host genome to capture useful sequences derived from retrotransposons.

### 4.2. Restriction of Ty1′ by an Endogenized Gag

*DRT2* itself consists of an approximately 1 kb segment derived from Ty1′ *GAG*. Examination of the fine structure of the locus in the wild Malaysian strain UWOPS05-227.2 (which contains *DRT2* but lacks full-length Ty elements in the genome) reveals the intact ORF that produces the inhibitory protein Drt2 [72]. There is a 1 bp deletion that results in multiple in-frame stop codons in the 5′ region of *GAG* but an ORF coding for the complete p22-equivalent region is retained, including the CA-CTD region of p18m. The *DRT2* ORF contains both the AUG1 and AUG2 translation start sites present in p22. All strains that contain *DRT2* maintain an intact ORF and analysis of synonymous and non-synonymous point substitutions provides evidence that it is evolving as a coding region under purifying selection [72].

Transcription of this locus produces an abundant transcript that initiates just upstream of the *DRT2* ORF from an internal start site similar to that of Ty1i [72]. A less abundant transcript was also detected that initiates from the TATA box of an upstream Ty2 solo LTR. Endogenous Drt2 protein levels were below the limit of detection by western blotting but a custom antibody raised against the 169 amino acid Drt2 of UWOPS05-227.2 (commercially available from Boster Bio cat. no. DZ41275) can detect Drt2 overexpressed under the *GAL1* promoter on a plasmid [72].

A series of genetic and biochemical studies demonstrated that Drt2 functions as a restriction factor of Ty1′ using a similar mechanism as p22/p18 does against Ty1c. Knockout of *DRT2* from chromosome IV in multiple *S. cerevisiae* strains resulted in an approximately four-fold increase in the retromobility of Ty1′ present on a centromere-based plasmid and expressed from its endogenous LTR promoter [72]. Furthermore, Drt2 proteins taken from across the *S. cerevisiae* phylogeny inhibited Ty1′ using a plasmid co-expression system. Similar to p18m, the CA-CTD region of Drt2 was shown to be the functional portion of the restriction factor; this minimal region (Drt2m) was sufficient to restrict Ty1′. Also like p22/p18, Drt2 co-fractionated with VLPs when yeast cell lysates were ultracentrifuged through a sucrose gradient, implicating a similar mechanism of restriction through a binding interaction between Drt2 and Ty1′ Gag [72] (Figure 5).

## 5. Evolution of Restriction of Ty1 Subfamilies

### 5.1. Evolutionary Loss of Host Transposon Control Systems

*S. cerevisiae* lacks several of the transposon control systems found in other eukaryotes such as RNAi or the restriction factors APOBEC, SAMHD1, and MOV10. While those restriction factors never evolved in yeast, the ancestor of *S. cerevisiae* lost RNAi machinery likely due to its incompatibility with the dsRNA killer viruses [123,124]. Killer viruses are found in many strains of *S. cerevisiae* and all its closely related species (the sensu stricto clade) except *S. kudriavzevii* [124,125]. These consist of a dsRNA virus, L-A, and its satellite dsRNA, M, that together maintain a killer toxin-producing and toxin-immunity phenotype [126,127,128]. Loss of RNAi in the ancestor of the sensu stricto clade and acquisition of killer, therefore, can provide a selective advantage for these species over nonkiller/toxin-sensitive neighbors. The absence of RNAi and other transposon control mechanisms may have selected for the expression of p22 and the exaptation of Drt2 restriction factors. Indeed, the introduction of RNAi components Dicer and Argonaute from another budding yeast of the same genus outside the sensu stricto clade, *S. castellii*, silences Ty1c elements in *S. cerevisiae* [123]. Consistent with this model, there has not been an active retrotransposon identified in the *S. castellii* genome, despite an abundance of Ty fragments and a large fraction of siRNAs corresponding to Ty sequences [123].

### 5.2. The Evolutionary Arms Race Between Retrotransposon and Restriction Factor

The Ty1c restriction factor p22/p18 is self-encoded and therefore all p22 sequences are present in Gag. This constrains an “arms race” between Gag and its restriction factor. Any mutations in the interfaces between p22 and Gag that may allow for CNC escape will simultaneously change p22 and Gag sequences and may have an impact on Gag function. Indeed, several CNC-resistant mutations in Gag result in lower Ty1c fitness [70]. Likely due to this constraint, the surfaces used by p18m to bind and inhibit Gag are extremely well conserved [68,70,71].

Unlike p22/p18, Drt2 is an exapted sequence from an ancient Ty1′ insertion. It is present in a locus that has been a hotspot of Ty activity and, while the original Ty1′ insertion has decayed, the *DRT2* ORF has been retained and selected for [72]. *DRT2* and Ty1′ are, therefore, able to accumulate mutations separately from one another. Nonetheless, Drt2m and the equivalent region in Ty1′ Gag are 91.5% identical containing only 9 amino acid differences, 3 of which are conservative substitutions (T274N, K322R, K354R). Furthermore, Drt2m can functionally replace the Ty1′ Gag CA-CTD with only a modest decrease in retromobility, indicating that Drt2 has retained considerable capsid functionality after its endogenization [72].

### 5.3. Restriction Specificity Between Ty1 Subfamilies

Ty1c and Ty1′ are closely related subfamilies (91.3% amino acid identity in Gag-Pol and 78.9% identity in Gag) [71] and given the similarities between p22/p18 and Drt2 restriction, despite their expression from different sources, it is reasonable to wonder if p22/p18 could inhibit Ty1′ or if Drt2 could inhibit Ty1c. However, chromosomal Drt2 showed no restriction activity against endogenously expressed plasmid-borne Ty1c [71], whereas in the same expression system Drt2 reliably lowers Ty1′ retromobility by approximately four-fold [71,72]. Also, in plasmid co-overexpression experiments, cross-restriction was either extremely weak or not statistically significant, demonstrating that p18m and Drt2m are highly specific to their own Ty1 subfamily [71].

Ty1c/p18m, Drt2m, and the equivalent CA-CTD region in Ty1′ Gag are each well conserved with over 90% amino acid identity within each group. But there are 17 amino acid differences between p18m and Drt2m. The crystal structures of p18m [68] and Drt2m [71] reveal that three of these divergent residues are found within Dimer-1 (Figure 4). Ty1c/p18m contains A266, V270, and L312 (abbreviated AVL) whereas Ty1′ and Drt2m contain V266, T270, F312 (abbreviated VTF), and each residue is 100% conserved within the respective subfamily. The two restriction factors are nearly structurally identical, but the AVL/VTF residues present a mechanism to discriminate between the Dimer-1 surfaces of the different subfamilies. The presence of added steric bulk from the divergent residues V266 and F312 in Ty1′ and Drt2m causes an invariant residue R315 to adopt an alternate rotamer, subtly changing the Dimer-1 surface from Ty1c/p18m. Additionally, the divergent T270 in Ty1′ and Drt2m can hydrogen bond with R315; this creates two new hydrogen bonds in the homotypic Drt2m/Ty1′ pairing because the two opposing CA-CTD monomers are antiparallel and each participates with T270 and R315 (Figure 4). Thus, the combination of this arginine-flip mechanism and the distinct hydrophobic surface created by the divergent three residues only support high-specificity homotypic pairings [71].

This explains the exquisite specificity that has evolved to precisely target each restriction factor to its own retrotransposon. Mutation of AVL/VTF is sufficient to severely abrogate restriction measured using retromobility assays with plasmid co-overexpression in vivo or homodimer formation by SEC-MALLS in vitro [71]. Moreover, p18m can be retargeted against Ty1′ by mutating its AVL residues into VTF, effectively creating a Drt2m/Ty1′ Dimer-1 surface. The opposite swap in Drt2m retargets it to restrict Ty1c. The same mutations were made in Gag as well and all pairwise combinations corroborated using the in vitro homodimer assay and the in vivo retromobility assay: a compatible Dimer-1 interface is necessary and sufficient for restriction [71].

### 5.4. Evolutionary Trajectories of Ty1c and Ty1′

Ty1′ is the ancestral element relative to Ty1c: the Ty1c subfamily arose due to a horizontal gene transfer of a *GAG* gene from *S. paradoxus* [61] (Figure 1). Ty1c was designated as canonical due to its abundance in the S288C genome and higher retrotransposition activity that allowed it to be captured on a plasmid and studied [52,54]. However, it is the younger element and is an intrinsically more active retrotransposon. Ty1c retromobility is an order of magnitude higher than Ty1′, whether expressed from its own LTR-promoter or overexpressed from the *GAL1* promoter, possibly due to different propensities to multimerize during the VLP assembly pathway [71]. Drt2 was endogenized early in the diversification and spread of global *S. cerevisiae* lineages [72] but Ty1c likely would not have been subject to Drt2-based restriction. The horizontal transfer of *GAG* may have included sequences that permit Ty1i expression and so p22-based restriction limited Ty1c retromobility once sufficient genomic copies were present. Ty1c, which is further divided into Ty1c1 and Ty1c2 variants, is the most abundant element but is limited to certain strains, consistent with a relatively recent divergence and rapid spread [55]. Ty1′ is more widely distributed but is present at a lower copy number [55,61].

Despite evolving along independent trajectories, the p22/p18 and Drt2 restriction factors converged on a common lattice-poisoning mechanism to inhibit VLP assembly of their respective retrotransposons. Furthermore, they use the same Dimer-1 binding interface to interact with the corresponding CA-CTD surface of Gag. Subtle differences in the amino acids of the Dimer-1 surface exclude heterologous pairing between subfamilies. The distinct evolutionary histories of Ty1c and Ty1′ that resulted in different amino acids in Dimer-1 created a subfamily barrier that neither restriction factor has overcome.

Ty1c and Ty1′ are found together in many strains [55,61] and it will be interesting to continue to study interelement competition, whether that is conflict based on restriction factors or otherwise. In addition to Ty1c and Ty1′, there is also the Ty101 subfamily which is found predominately in South American strains [55]. Ty2 is the most abundant and widely distributed retrotransposon family in *S. cerevisiae* [55] and experiments with a model retrotransposition-competent Ty2 element (Ty2-917) suggest that it is not under a p22-like CNC mechanism. Ty2-917 did not confer restriction against Ty1c nor was Ty2-917 sensitive to p22 [70]. Multiple subfamilies of Ty1 have Ty2 introgression indicating a history of interactions while sharing the same host genome [55,61]. Ty3, Ty4, and Ty5 contain fewer distinct subfamilies than the Ty1 family, are present at far lower abundance across *S. cerevisiae*, and each have gone extinct in many isolates [55]. Transposon control mechanisms have not been investigated yet in these other elements, or in the related LTR-retrotransposons of *S. paradoxus* or other budding yeasts.

## 6. Summary and Outlook

The *S. cerevisiae* Ty1 family of LTR-retrotransposons has been a fertile system to study retrotransposon replication and restriction. In lieu of the transposon control mechanisms used in many mammals, yeast has evolved a mechanism to restrict the Ty1c and Ty1′ subfamilies by interfering with VLP assembly using CA-CTDs expressed either from internally initiated Ty1i or the endogenized gene *DRT2*. Each restriction factor is precisely targeted to its respective transposon using shared Dimer-1 surfaces. Further study of the mechanism and consequences of this restriction will continue to advance transposon biology. For example, it remains uncertain how Gag CNC-resistance mutations that cluster in the CA-NTD allow for escape from homotypic binding of p18m through the Dimer-1 surface in the CA-CTD. Furthermore, detailed investigation of the stoichiometry and kinetics of lattice-poisoning may provide insights about past and ongoing evolutionary conflicts. Analysis of other yeast LTR-retrotransposons may reveal additional novel mechanisms of restriction.

The p22/p18 and Drt2-based restriction is conceptually similar to a number of Gag-like restriction factors that protect against retroviral infection. In humans, endogenous HERV-K Gag co-assembles with HIV-1 and interferes with viral replication [129,130]. Similar to the lattice-poisoning by p18m and Drt2m, HERV-K appears to interfere with the early stages of virus assembly and mutation of residues of the HIV-1 CA-CTD involved in dimerization can provide resistance to HERV-K restriction. In sheep, co-opted retroviral Gags restrict the oncogenic retrovirus JSRV by forming heterocomplexes that ultimately lead to degradation of incomplete viral particles [131,132,133]. There have been amplification and gene conversion events of the endogenized Gag sequences throughout sheep domestication [134] offering interesting parallels to the highly dynamic *DRT2* locus. In mice, the endogenized retroviral Gag gene *Fv1* [135,136] protects against MLV [137] and, similar to the specificity of p22/p18 and Drt2, certain alleles, *Fv1^b^* and *Fv1^n^*, confer resistance to MLV of the opposing N and B tropism [138,139,140]. The mechanistic details elucidated by studying Ty1 may continue to provide insights to the restriction of other retroelements as continued study may reveal additional examples related to the Ty1 restriction system.

The lattice-poisoning mechanism used by p22/p18 and Drt2 suggests a new target for pharmacological inhibition, although care would need to be taken to not interfere with host fitness as engineered Drt2m-AVL appears to have a cytotoxic effect in yeast in certain expression contexts [141]. Other endogenous LTR-retrotransposons or exogenous viruses that require particle assembly for their replication may be vulnerable to inhibitors that target similar binding surfaces along the particle assembly pathway.

## Data Availability

No new data were created in this study.

## Abbreviations

The following abbreviations are used in this manuscript:

LTR	Long terminal repeat
VLP	Virus-like particle
CNC	Copy number control
CA	Capsid
NTD	N-terminal domain
CTD	C-terminal domain
PR	Protease
RT	Reverse transcriptase
IN	Integrase
